# An Elementary Treatise on Midwifery; or Principles of Tokology and Embryology

**Published:** 1846-03

**Authors:** 


ART. VIII.—*An Elementary Treatise on Midwifery; or Principles of Tokology and Embryology.* By ALF. A. S. M. VELPEAU, M. D., &c. &c. Translated from the French, by CHARLES D. MEIGS, M. D., Member of the American Philosophical Society; Professor of Midwifery in the Jefferson Medical College, &c. Third American edition. With *Notes and Additions.* By WILLIAM HARRIS, M. D., Member of the American Philosophical Society; Lecturer on Midwifery aud the Diseases of Women and Children, &c. Philadelphia: Lindsay & Blackiston. 1846. (8vo. pp. 800.)

This work has been well received by the profession in America, as is shown by the circumstance that it has passed to a third edition. The name of Velpeau is familiar to all medical readers. As a writer, he is voluminous beyond any living medical man, having, it is said, written for the press more than twenty-five thousand pages, amounting to seventy-two volumes; and his age still does not exceed fifty years. Besides these medical works, he is the reputed author of one hundred and forty other articles and memoirs. His industry is wonderful, and he seems to be acquainted with all that has been contributed to medical literature in his own and every other language. The work before us possesses peculiar merits as an elementary treatise on midwifery, and may be profitably placed in the hands of students. Whatever was originally deficient in it has been supplied by the learning and judgment of our countrymen, who have translated and edited the work. Among these additions one of the most elaborate and valuable is a chapter, by Dr. Harris, on *puerperal fever.*

